# A Flexible Flow Sensor System and Its Characteristics for Fluid Mechanics Measurements

**DOI:** 10.3390/s91209533

**Published:** 2009-11-27

**Authors:** Peng Liu, Rong Zhu, Ruiyi Que

**Affiliations:** State Key Laboratory of Precision Measurement Technology and Instruments, Department of Precision Instruments and Mechanology, Tsinghua University, Beijing, 100084, China; E-Mails: liupeng07@mails.tsinghua.edu.cn (P.L.); katykob@163.com (R.Q.)

**Keywords:** hot-film flow sensor, flexible printed circuit board, flow measurement

## Abstract

In this paper, we present a novel micromachined hot-film flow sensor system realized by a technique using a film depositing processes and incorporating a standard printed circuit. Sensor electrodes and electronic circuits are preprinted on a flexible substrate of polyimide (PI), *i.e.*, a flexible printed circuit board (FPCB). The sensing element, which is made of Cr/Ni/Pt with a temperature coefficient of resistance around 2,000 ppm/K, is fabricated on the FPCB by either magnetron sputtering technology or pulsed laser deposition (PLD). The sensor can be packed efficiently at high-density and integrated with signal processing circuits without additional pads. A simple fabrication process using mature technique and materials selection guarantees that the time and costs are greatly reduced. Both steady-state and transient characteristics of the sensors are experimentally tested, and the results presented to validate the effectiveness of the sensors.

## 1. Introduction

The measurement of fluid mechanics is very important in various industrial fields, and flow sensors have been widely applied to execute accurate and efficient measurements. Of these sensors, micromachined flow sensors have been developed for several decades. The silicon-based flow sensor was first demonstrated in 1974 [1] and since then many application-oriented flow sensors have been developed based on various sensing principles, such as thermal anemometry [1], Doppler frequency shift, and indirect inference from pressure differences [2-4]. Among these sensors, thermal flow sensors possess merits of simple structure and easy use, and thus offer practical solutions for various fluidics applications [3].

Hot-wire and hot-film anemometers utilize a thermal element that serves as both a Joule heater and a temperature sensor. Under a constant bias power and zero flow rate, the temperature of the thermal element reaches a steady-state value. If an external fluid flow is presented around the thermal element, the thermal element experiences forced convective cooling. Accordingly, the temperature of the thermal element provides the means to gauging the cooling rate and the flow velocity [4].

The adoption of microfabrication for sensors has shown many technical and economical advantages compared with other conventional fabrication technologies [5]. Microfabrication offers the benefits of higher performance and functionality, at lower cost, with decreased size and increased reliability. Moreover, definition and reproduction of the device shapes are achieved using photolithography techniques which fundamentally have very high precision so that it is effective to make large arrays of sensors with good uniformity [6]. In the past two decades, several research groups have demonstrated micromachined flow sensors based on various principles, including thermal transfer [7], torque transfer [8], and pressure distribution [9]. In addition to flow speed sensors, boundary-layer shear stress sensors have been realized using floating element methods [10] and thermal transfer principles [11].

Currently, most existing micromachined sensors have been developed using single-crystal silicon substrates. An important reason for making sensors out of silicon lies in the fact that piezoresistive element can be realized in silicon by selective doping. However, silicon devices are relatively expensive and brittle when compared with polymer and metal-based devices. A silicon beam may fracture easily under shock or contact. Besides, the lead bonding between the sensor and the signal conditioning circuit is complicated to operate. In recent years, flow sensors on polyimide substrates have been also demonstrated [2,12,13]. These sensors have the advantages of low cost and flexibility. However, the sensor structure is still complex and fragile without including an electric circuit on a chip. In this paper, we present a novel flow sensor using a flexible polyimide substrate, which is fabricated by integrating a standard flexible printed circuit technique with thin-film sputtering/deposition. The advantages of these sensors include simple structure, low-cost, and flexibility, thus being easy to attach on object surfaces, integrating good mechanical characteristics and sensing capability with electric circuits. The steady and dynamic characteristics of the sensors and sensor system are systematically tested and the application prospects are analyzed.

## 2. Sensor Design and Fabrication

### 2.1. Sensing Principle

The working principle of thermal flow sensors relies on the detection of the convective heat transfer between an electrically heated resistive sensing element (hot-wire or hot-film) and fluidic flow. The hot-wire or hot-film experiences cooling due to heat transfer given that the sensor is heated to a higher temperature than that of the surroundings. Heat transfer depends on the flow velocity. Thus flow sensing is accomplished without using any moving parts and requires only thermal elements that serve as both Joule heaters and temperature sensors. The thermal flow sensor is small, with high spatial resolution, simple to implement and easy to array locally.

The flow field information around the surface of an object could be divided into two components, that is, the normal pressure exerted on the surface and the shear stress along the surface. Both of the components are useful for inferring the flow parameters, such as flow velocity [14,15]. We measure the flow velocity that is correlative with both the normal pressure and the shear stress by using hot-film flow sensors, which will be described in the following section. In our measuring mode, sensors are located in or near a flow environment and experience heat loss when subjected to fluid flow. Flow sensing is accomplished by monitoring the resistance change with temperature.

### 2.2. Substrate and Sensing Elements

We choose polyimide as substrate because the thermal conductivity of polyimide is low, almost two orders of magnitude lower than that of silicon. Polyimide also has good mechanical properties, can be patterned by oxygen plasma, and is chemically inert. In addition, it is a standard material of flexible printed circuit boards, so the sensor electrodes and circuits can be fabricated using a standard flexible printed circuit technique. For the above considerations, the selection of polyimide provides both technical and economical advantages. In this work, the thickness of the polyimide substrate is roughly 150 μm due to standardization concerns. After testing, we realized that this thickness can satisfy the requirement of mechanical rigidity and will not be too thick so as to decrease the frequency response of the hot-film sensor due to dramatically added thermal mass.

**Figure 1.**
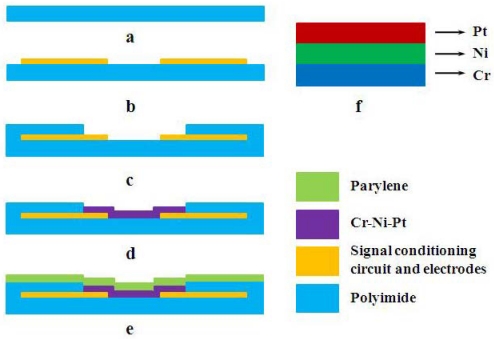
Fabrication process of hot-film sensor. (a–c) Standard printing technique on flexible printed circuit board. (d) Deposition of Cr-Ni-Pt thin-film. (e) Deposition of parylene. (f) The structure of the composite thin-film layer.

Conventional hot-wire/hot-film sensors adopt tungsten, platinum, or metal alloys as the sensing element. Tungsten has a high-melting point and is difficult to deposit. Platinum (Pt) does not have as high a temperature coefficient of resistance (TCR) value as nickel (Ni), but the oxidation resistance behavior of Ni is not satisfactory, while Pt has good antioxidation properties. Considering both sensitivity and stability, the thermal elements of our hot-film sensors are made of composite materials. Ni is used as the main thermal element material, Pt as a cover layer and chromium (Cr) as a bonding layer between Ni and the substrate PI for enhancing the adhesion of the sensing element [Figure 1(f)].

### 2.3. Fabrication

**Figure 2.**
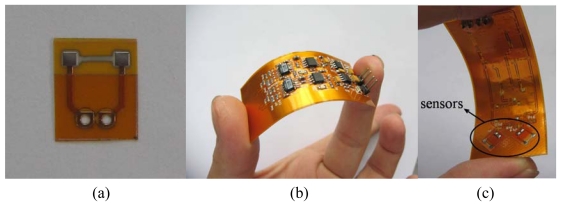
Sensors with signal conditioning circuit: (a) a sensor unit (7 × 8 × 0.02 mm); (b) the reverse side of a measurement system with the circuit (20 × 60 × 1 mm); (c) the front side of the system with two sensors.

The film of thermal elements can be deposited by either magnetron sputtering technology or pulsed laser deposition (PLD). In this paper, we take the magnetron sputtering technology as an example to demonstrate the fabrication process. The overall fabrication process is shown in Figure 1. The process starts on a FPCB with a standard printing technique [Figures 1(a–c)] and the sensor electrodes and signal conditioning circuit (Cu/Au) are thus printed on the FPCB. The prepared electrodes and circuit help to solve the complicated lead bonding problem. Then we deposit the metal composite thin-films [Figure 1(d)], orderly from bottom to top, a 40-nm-thick chrome serving as an adhesion layer, a 160-nm-thick nickel forming the main thermal element, and a platinum layer with a thickness of approximately 30 nm used as a protection. The metal film is patterned to be 3 mm long and 0.3 mm wide and lie across two electrodes by using a prepared shadow mask. The sputtering of the metal thin-film is carried on in a vacuum of 1 × 10^–4^ Pa, with a power of 80 W. Finally an around 200-nm-thick parylene film is deposited as encapsulation as shown in Figure 1(e). Figure 2 shows a fabricated sensor unit and a sensor system with two sensors on the one side of FPCB [Figure 2(c)] and a signal processing circuit on the other side [Figure 2(b)]. When combining multi sensors to form an array system, the distances between the sensors need to be well-designed to prevent any interference from each other.

### 2.4. Post Treatment

**Figure 3.**
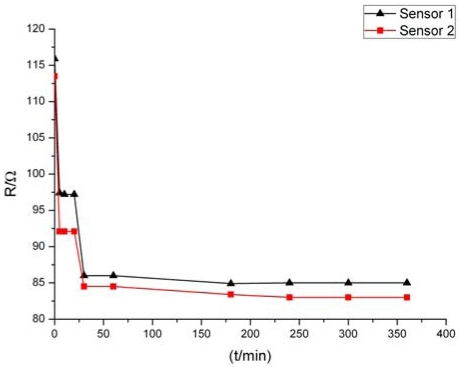
Changes of sensor resistance during electrifying based on self heating.

After the fabrication of the sensor structure, a post treatment is performed in order to make the sensors more stable and durable. Conventionally, the sensor is annealed at a temperature about 100 °C above recrystallization temperature of the material in a heat processing step. Considering the heat resistance of the PI substrate, we heated the sensor at 200 °C for 1 hour in a vacuum environment. Then the sensors are further electrified for aging with a 30 mA current for 3 hours based on self heating. Through these post treatments, the structural defects and internal stress are eliminated, and accordingly, the stability and TCR of the sensors are enhanced. We found that the linearity relation between the resistance and the temperature of the sensor is improved by the heating process and the stability of the sensor is advanced by the electrifying. Figure 3 shows the effect of the electrifying (self heating). It can be seen that the resistance of the sensor changes as the electrified time increased. Within 1 hour, the resistance changes dramatically. After 3 hours, the resistance tends to become stable. It is also found that the TCR of the sensor can be almost doubled via electrification. The TCR of the successful sensors is finally tested to be about 2,000 ppm/K. The failure probability of the finished sensors in the fabrication is less than 10%, mainly due to nonuniformity of the substrate material and sputtering film. The resistance and TCR varied less than 0.1% per day over the long term measurement.

## 3. Sensor Characteristics

### 3.1. Sensor Calibration

**Figure 4.**
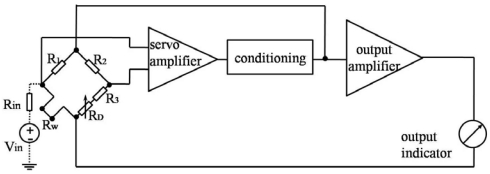
Schematic view of constant temperature circuit: *R*_2_/(*R*_3_ + *R_D_*) = 1/2, *R_in_* ≫ *R*_1_, *R_in_* and *V_in_* are only used for dynamic testing.

A hot-film/hot-wire sensor can be operated either in constant voltage (CV), constant current (CC) and constant temperature (CT) modes. In CV mode, the heating voltage is held constant, for which the circuit is easy to be realize but the sensitivity is low. In CC mode, the heating current, or power, is held constant and the sensitivity and the response are low. Conversely, in CT mode, the sensor temperature is held constant by feedback circuitry and the increase in heating power required for maintaining constant temperature is measured in response to flow. Feedback provides automatically adjusting electronic compensation for the thermal inertia of the filament as its operating point varies. The major advantage of CT mode is that the frequency response is quicker and the sensitivity is higher than that of other modes [16]. In this paper, we use the CT mode shown in Figure 4 to operate the hot-film sensors for flow measurements.

Steady-state output of an infinitely long hot-wire follows King's half power law [15], which states that:

(1) $$Nu=A+B\sqrt[{n}]{R}$$

where Nu and R are the Nusselt and Reynolds number, respectively, and A and B are constants, which can be determined via calibration.

For realizing the measurement of the sensors, the hot-film sensor is placed on one leg of a Whetstone bridge as *R_W_* The bridge circuit is balanced when *R*_1_(*R*_3_ + *R_D_*) = *R_W_R*_2_. The resistance *R*_3_ is a temperature compensating resistance and the resistance *R_D_* is an adjustable resistance used to set the overheat ratio *α* using the following equation:

(2) $$R_{3}+R_{D}=\frac{R_{2}R_{W}}{R_{1}}(1+\alpha )$$

The overheat ratio *α* dominates the sensitivity of the sensor and also determines the power comsumption. With the increase of *α*, the sensitivity increases but the power consumption increases as well. A well tradeoff needs to be balanced.

**Figure 5.**
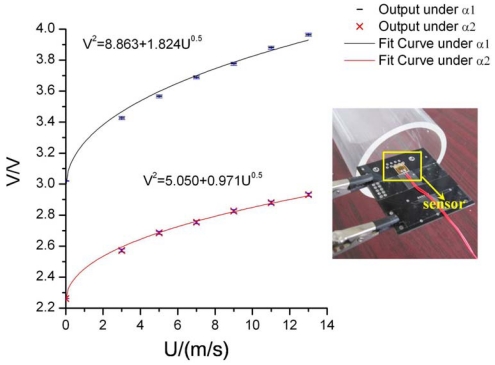
Measurement results of the sensor with error bars under constant temperature mode. Bridge ratio of the CT circuit is 20, and overheats ratio α1 and α2 are set as 0.03 and 0.02 respectively.

The term *R*_2_/*R*_1_ is called the bridge ratio. It can be shown that the voltage output *V* varies with flow speed *U* shown as follows:

(3) $$V^{2}=C+DU^{n}$$

where *C* and *D* are constants that can be determined through experimental calibration. [17] We have done the wind tunnel experiment. The fabricated sensor is attached to the edge of a PC board (Figure 5). The experimental output of the sensor under CT mode is shown in Figure5. The solid lines are fitted by using the theoretical model (4) where n is assumed as 0.5 [17]. From the measurements, the sensitivity of the sensor is estimated to be around $1.8V^{2}/\sqrt{\left(m/s\right)}$ and $0.9V^{2}/\sqrt{\left(m/s\right)}$ at the overheat ratio of 3% and 2% respectively. The results validate again that the sensitivity of the sensor increases as the overheat ratio increases.

Considering that the resistance and TCR of sensors may change when the sensors are attached on different curved surfaces, we tested the effects of curvature. When the surface curvature changes in the range of 0–50, the resistance and TCR change also, but less than 2% and 5%, respectively, which could be compensated by a calibration beforehand. For an accurate measurement, we assume that the sensors will be attached on smooth surfaces with curvatures as small as possible.

### 3.2. Dynamic Response

We further tested the time constant of the sensor under the CT mode. Because of the difficulty in generating a pulsed or step flow velocity profile with sharp transient, a small signal square wave *V_in_* supply is injected into the circuit to act as the disturbance [16]. The square wave test assumes varying the heating current can represent the heating and cooling of the hot-film with velocity fluctuation. For example, at the onset of a voltage increase, the feedback circuit will try to balance the bridge by decreasing the voltage output and hence the heating current; this is similar to a sudden decrease in fluid velocity. The time it takes for Vout to settle back to 3% of peak value is defined as *τ_w_*, and the response time can be calculated by *τ_c_* = 1.3*τ_w_* [18].

**Figure 6.**
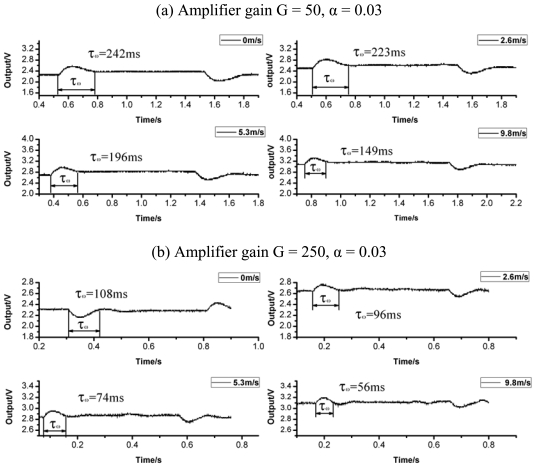
Time response curves under different flow vlocities. (a) Amplifier gain G = 50, α = 0.03 (b) Amplifier gain G = 250, α = 0.03

The time responses under different flow velocities were tested and recorded as shown in Figure 6 and listed in Table 1. From the data, it can be concluded that the time constant becomes shorter as the flow velocity increases and can be adjusted by tuning the circuit parameters, such as amplifier gain (G). The hot-film sensor feedback system can be regarded as a semi-second-order system. The overshoot and the response speed are two conflictive parameters in the system. A tradeoff must be achieved here according to the application requirements.

### 3.3. Directional Sensitivity

**Figure 7.**
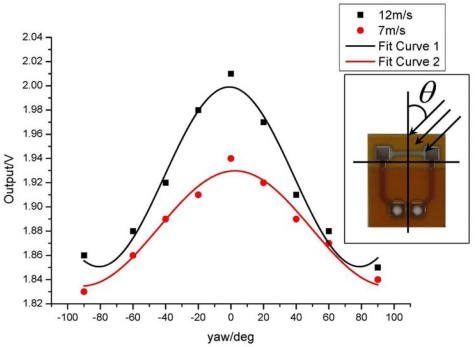
Angular responses under different flow velocities.

Due to its strip profile, the hot-film sensor is also sensitive to the direction of fluid flow. Under a sufficiently large aspect ratio, the yaw response of the hot-film sensor follows the cosine relation, namely [17]:

(4) $$U(\theta )=U_{0}\cos(\theta )$$

where *θ* refers to the yaw angle between the flow direction and the sensor axis illustrated in Figure 7.

We tested the angular response of the sensor at the angle range of −90° ∼ 90° under the CT mode. The output voltages at different flow velocities are shown in Figure 7, where solid lines are fitted by the cosine law.

The testing angular response of the sensor deviates slightly from the cosine law due to the low aspect ratio for our sensors (typically 10). Under the low aspect ratio, the sensor suffers a tangential cooling effect. However the measurement results still exhibit a directional dependence, which can be applied to gauge the flow vector by using sensor arrays [19,20].

### 3.4. Characteristic Analysis and Applications

The experimental performance of our prepared sensor system under CT, including noise density and electrical parameters, are summarized in Table 2. The electrical parameters shown in Table 2 indicate that the sensor system is operated with low power. The power supply for the sensor system could rely on a standard battery. Many applications requiring sensors and the corresponding systems are highly compact, portable and durable. It has been tested that our prepared sensor system can work continuously for more than 10 hours with the power supplied by a standard 7.4 V–600 mAh lithium battery. However, the time response of the hot-film sensors is not fast as conventional hot-wire sensors (typically in the order of kHz). Therefore, the application of the sensors is limited to fluidic measurement in a low frequency, e.g., flight parameters measurements for a micro air vehicle [21]. The sensitivity, flow rate range, and frequency response can be further enhanced by increasing the overheat ratio of the sensor at the expense of power economy. From the view of the economical reason and shorter production period, the sensors can be adopted in harsh and high-loss situations. In some applications with high-integrated requiements, these hot-film sensors are preferred to others since the conventional complicated lead bonding required in the packaging of the sensor have been simplified by PCB welding. In addition, sensor array system can be easily realized on the FPCB and mass-produced economically.

## 4. Conclusions

We present a novel hot-film flow sensor with a flexible substrate. The sensor is fabricated directly on a flexible printed circuit board, which is economical and easy to integrate with a signal processing circuit. As a result, hot-film anemometry sensors can be potentially produced with low cost and high fabrication efficiency. Fluid mechanics measurements can also be further realized by using large arrays of these sensors. The sensor system has the advantages of good mechanical characteristics and sensing capability, simple structure, low-cost, and flexibility, thus being easy to attach on object surfaces.

## Figures and Tables

**Table 1.** Response time under different conditions (corresponds with *τ_c_* = 1.3*τ_w_*).

Flow velocity / (m/s)	0	2.6	5.3	9.8
Time constant *τ_c_* of (a) / ms	314.6	289.9	254.8	193.7
Time constant *τ_c_* of (b) / ms	140.4	124.8	96.2	72.8

**Table 2.** The performances of the flow sensor system.

Flow rate range	0–15 m/s (α = 0.03)
Angular sensitivity range	0–60°
TCR	2,000 ppm/°C
Inaccuracy	±3% F.S.
Repeatability	±0.3% F.S.
Response time	*τ_c_* = 72.8 *ms* (*G* = 250, α = 0.03, 9.8 m/s)
Zero adjustment	Overheat ratio dependent
Supply voltage	8*V_DC_* ± 10%
Output signal	1.5 V–4.5 V
Resolution	0.1 m/s
Power consumption	30 mW(*G* = 50, α = 0.03, 5 m/s)
